# Malawi Mental Health Guide: Overview and Evaluation of a Mental Health Quick Reference Guide and Phone App for Use in Non-specialist Settings

**DOI:** 10.1192/bjo.2024.89

**Published:** 2024-08-01

**Authors:** Donncha Mullin, Kazione Kulisewa, Owen Mwale, Rui-Shian Lee, Rob Stewart

**Affiliations:** 1 University of Edinburgh, Edinburgh, United Kingdom; 2 NHS Lothian, Edinburgh, United Kingdom; 3 Scotland-Malawi Mental Health Education Project, Edinburgh, United Kingdom; 4 Kamuzu University of Health Sciences, Blantyre, Malawi; 5 Malawi Epidemiology and Intervention Unit, Lilongwe, Malawi

## Abstract

**Aims:**

In Malawi, there are three Consultant psychiatrists for a population of approximately 20 million people. We cannot rely solely on specialists to provide mental health care. We produced the Malawi Quick Guide to Mental Health (the Guide) to improve the psychiatric health-care resources available to frontline mental health workers in Malawi, thus improving service provision to patients. We aimed to evaluate its impact on the frontline non-specialists who provide most mental health care in Malawi.

**Methods:**

In collaboration with the Malawi Ministry of Health, the University of Malawi, St John of God Malawi, and a Malawian user group, a group of psychiatrists with experience working in Malawi co-produced the Malawi Quick Guide to Mental Health. It provides practical information for assessing and managing mental disorders in Malawi. We distributed the Guide to over 400 health centres in Malawi. Next, we converted the Guide into a freely available phone app in both Android and Apple stores.

To study its impact, we baseline surveyed frontline mental health professionals regarding their access to basic psychiatry guidelines and information in clinics, as well as their confidence in delivering mental health care. We repeated this survey six months after the distribution of the printed Guide and six months after the app launch.

**Results:**

Baseline survey: 20 health-care professionals representing regions throughout Malawi responded. 70% of respondents were between 25–40 years old and 45% were female. All respondents either agreed or strongly agreed that they needed more support caring for mentally unwell patients. 15% had no access to any resources whatsoever to guide their care.

Printed guide survey: 95% agreed or strongly agreed that having a printed copy of the Guide increased their confidence in caring for patients. Information resource accessibility, availability and usage in mental health clinics had improved from baseline. The respondents found the Guide helped their day-to-day practice, with 95% rating it either extremely helpful or very helpful. 95% either agreed or strongly agreed that it had improved the care they provided their patients.

App survey: 66% of respondents prefer using the app over the textbook version. All agreed that the app made them more confident in caring for their patients and that their care had improved because of the app. They were all likely to recommend it to a colleague. It has now been downloaded almost 1000 times.

**Conclusion:**

A free, co-produced mental health book and phone app have helped to address the issue of limited access to basic psychiatry guidelines and information in clinics in Malawi. This has improved clinicians’ confidence and their perceived patient care.

